# Research progress of spontaneous ruptured hepatocellular carcinoma: Systematic review and meta-analysis

**DOI:** 10.3389/fonc.2022.973857

**Published:** 2022-09-29

**Authors:** Chunling Wang, Xiaozhun Huang, Xiaofeng Lan, Dongmei Lan, Zhangkan Huang, Shu Ye, Yihong Ran, Xinyu Bi, Jianguo Zhou, Xu Che

**Affiliations:** ^1^ Department of Hospital-Acquired Infection Control, National Cancer Center/National Clinical Research Center for Cancer/Cancer Hospital & Shenzhen Hospital, Chinese Academy of Medical Sciences and Peking Union Medical College, Shenzhen, China; ^2^ Department of Hepatobiliary Surgery, National Cancer Center/National Clinical Research Center for Cancer/Cancer Hospital & Shenzhen Hospital, Chinese Academy of Medical Sciences and Peking Union Medical College, Shenzhen, China; ^3^ Department of Internal Medicine, National Cancer Center/National Clinical Research Center for Cancer/Cancer Hospital & Shenzhen Hospital, Chinese Academy of Medical Sciences and Peking Union Medical College, Shenzhen, China; ^4^ Department of Hepatobiliary Surgery, National Cancer Center/National Clinical Research Center for Cancer/Cancer Hospital, Chinese Academy of Medical Sciences and Peking Union Medical College, Beijing, China

**Keywords:** hepatocellular carcinoma, spontaneously ruptured, hepatectomy, survival, prediction model

## Abstract

**Background:**

Spontaneously ruptured hepatocellular carcinoma (rHCC) with hemorrhage is characterized by rapid onset and progression. The aim of this systematic review was to explore the current studies on rHCC with hemorrhage and determine the optimum treatment strategy.

**Method:**

The PubMed, Web of Science, Embase, and the Cochrane Library databases were searched for studies reporting survival outcomes with comparison between emergency resection (ER) and transarterial embolization following staged hepatectomy (SH) were included by inclusion and exclusion criteria, the perioperative and survival data were statistically summarized using Review Manager 5.3 software.

**Result:**

A total of 8 retrospective studies were included, with a total sample size of 556, including 285 (51.3%) in the ER group and 271 (48.7%) in the SH group. The perioperative blood loss and blood transfusion volume in the SH group were less than those in the ER group, and there were no significant differences in the operative time, incidence of complications, mortality and recurrence rate of tumors between the two groups. The 1-, 2-, 3-year overall survival and 1-, 2-, 3-, 5-year disease-free survival of the ER group were not significantly different from those of the SH group, and the 5-year overall survival rate of ER group was lower than that of the SH group (hazard ratios=1.52; 95% confidence intervals: 1.14-2.03, P=0.005).

**Conclusion:**

There was no significant difference in the short-term efficacy of ER or SH in the treatment of ruptured HCC, and SH was superior to ER in the long-term survival.

## 1 Introduction

Hepatocellular carcinoma (HCC) is the fifth most common malignancy worldwide and the second most common cause of cancer-related deaths (1). Spontaneous rupture is a rare but fatal complication of HCC that is characterized by coagulation disorders, hemodynamic instability, and hepatic insufficiency. Ruptured hepatocellular carcinoma (rHCC) is more common in patients with advanced liver disease and heavy tumor burden, which is reflected tumor size, number of tumors, portal vein cancer embolism, and microvascular invasion.

The current treatment strategies for rHCC include emergency resection (ER), anhydrous alcohol injection, hepatic artery ligation, transcatheter artery embolization (TAE), and conservative symptomatic supportive care. Radical resection is a curative option for rHCC, and its goal is to stop bleeding in time and salvage liver function. However, given the poor general condition and liver function of patients with rHCC, the tumor is usually unresectable, large, or multifocal, and may be accompanied by major intrahepatic vascular invasion and extrahepatic metastases. This not only obviates the use of exploratory laparotomy for radical resection but also increases the risk of serious postoperative complications. TAE is superior to laparotomy in terms of maintaining hemostasis, and prolongs patient survival (2). Nevertheless, ER or TAE following staged hepatectomy (SH) is still a controversial treatment strategy for rHCC (3, 4).

According to the AJCC TNM staging, HCC with spontaneous rupture is classified as T4 stage regardless of primary tumor size and relationship to blood vessels (5, 6). However, some studies show that classifying all cases of rHCC as T4 may not accurately reflect the true prognosis (7–9). Therefore, it is critical to identify novel indicators or models to predict the prognosis of rHCC in order to guide clinical management. In this systemic review and meta-analysis, the research progress and prognostic models of spontaneous rHCC based on the available clinical evidence will be discussed.

## 2 Materials and methods

The present meta-analysis was performed according to the criteria defined by the Preferred Reporting Items for Systematic Reviews and Meta-analyses statement (10).

### 2.1 Databases and search strategies

The present meta-analysis was performed according to the criteria defined by the Preferred Reporting Items for Systematic Reviews and Meta-analyses statement. PubMed, Web of Science, Embase, and Cochrane Library databases were searched for articles that were available until February 25, 2022. Medical subject headings combined with free text words were used to search for randomized clinical trial (RCT) and observational studies. The following medical heading terms and their combinations were used: ((hepatocellular carcinoma [Title/Abstract]) AND (rupture [Title/Abstract])) AND ((hepatectomy [Title/Abstract]) OR (resection [Title/Abstract])).

### 2.2 Literature inclusion and exclusion criteria

Inclusion criteria (1): Published articles comparing the short and long-term outcomes of emergency resection (ER) or staged hepatectomy (SH) after hepatocellular carcinoma (rHCC) rupture; (2) Pathologically confirmed diagnosis of HCC in the study population; (3) Studies include at least one outcome measure relevant to the study.

Exclusion criteria: (1) Unextractable data; (2) Editorials, editorial letters, comments, case reports, or other types of publications; (3) Animal experiments.

### 2.3 Data extraction and outcome measures

After removing the duplicate articles, the titles and abstracts of the remaining articles were evaluated, and studies were sequentially excluded according to the eligibility criteria. The complete text of the selected articles was examined independently by two investigators, and any discrepancy was resolved by consensus. The main indicators included perioperative conditions (duration of surgery, amount of bleeding, amount of blood transfusion), postoperative outcomes (morbidity, mortality, recurrence rate), overall survival (OS), and disease-free survival (DFS).

### 2.4 Quality assessment and statistical analysis

The data were checked for completeness, plausibility, and integrity before incorporating them into a single database. The methodological quality of the retrospective studies was assessed using the modified Newcastle–Ottawa scale, which is based on patient selection, comparability of the study groups and outcome assessment. The studies were scored from 0–9, and scores ≥6 were considered high quality. Discrepancies, if any, were resolved by consensus. The meta-analysis was performed using the Review Manager 5.3 software (Cochrane Collaboration, Oxford, UK). Continuous and dichotomous variables were expressed as weighted mean difference (*WMD*) and odds ratio (*OR*) respectively. Results were reported with 95% confidence intervals (*CI*). Statistical heterogeneity among the included studies was assessed using the chi-squared test with *P* < 0.05 as the threshold of significance, and quantified using the *I^2^* statistic. The random effects model was used for pooled analyses in case of significant heterogeneity between the studies, and the fixed effects model was used otherwise. Bias in publication was tested using the Stata version 12.0 software (Stata Corporation, College Station, TX, USA).

## 3 Result

### 3.1 Search results

A total of 963 articles were initially retrieved and 366 duplicate studies were removed. The remaining 574 articles were screened on the basis of their titles and abstracts, and the irrelevant studies, case reports, and studies analyzing molecular mechanisms were excluded. The full texts of 23 articles were evaluated, and 8 articles (2–4, 11–15) were finally selected. The literature search and study selection criteria are schematically illustrated in **Supplementary Figure 1**.

### 3.2 Characteristics of the included studies

The characteristics of the 8 articles are summarized in **Supplementary Table 1**. All studies were retrospective, and published from 2006 to 2020. Except for the study by Buczkowski et al. (15) that was conducted in Canada, all studies were from China. The studies included 556 cases, of which 285 (51.3%) had ER and 271 (48.7%) had SH.

### 3.3 The methodological quality of the included studies

Based on the mNOS scores, the studies included in this meta-analysis were of high quality (**Supplementary Table 2**). Two studies (3, 4) scored 9, one study (12) scored 8, four studies (2, 11, 14, 15) scored 7, and only one study (13) scored 6 points. Four trials (3, 4, 12, 15) reported follow-up time, seven studies (2–4, 11, 12, 14, 15) reported perioperative outcome measures, and all studies reported survival data of at least 1 year.

### 3.4 Perioperative relevant outcome measures

Data of operation time was reported for the ER and SH groups in 4 studies (2–4, 15) using *WMD*. There was low heterogeneity among the studies (*I²*=5%), and the fixed-effects model showed no significant difference between the two groups (*WMD*=0.76 min, 95%*CI*: 9.28-7.76, *P*=0.86, **Supplementary Figure 2A**).

Five studies (2–4, 11, 15) reported perioperative blood loss and 4 studies (2–4, 15) reported blood transfusion, and the results showed that the ER group lost more blood than the SH group (*WMD*=683.61 mL, 95%*CI*: 283.36-1083.86, *P*=0.0008, **Supplementary Figure 2B**). Therefore, the need for blood transfusion was also significantly higher in the ER group (*WMD=*453.43 mL, 95%*CI*: 250.27-656.58, *P*<0.0001, **Supplementary Figure 2C**). Significant heterogeneity was observed for the blood loss results (*I*²=82%, *P*=0.0002), and moderate heterogeneity was found in the rate of blood transfusion (*I*²=49%, *P*=0.12). The sensitivity analysis reduced heterogeneity but do not change the statistical results.

Five studies (3, 4, 11, 14, 15) reported the incidence of perioperative complications, and the results showed that the complication rates of the ER and SH group were similar (23.6% vs. 40.9%; *OR*=0.94, 95%*CI*: 0.51-1.66, *P*=0.82, **Supplementary Figure 2D**), and there was no heterogeneity (*I^2 =^* 0). The incidence of postoperative liver failure was reported in 5 articles, and no statistically significant difference between the two groups (3.6% vs. 2.3%; *OR*=1.19, 95% *CI*: 0.40 to 3.57, *P*=0.75, **Supplementary Figure 2E**) or any heterogeneity among the studies was observed (*I^2 =^* 0).

Case fatality was reported in 6 studies (3, 4, 11, 12, 14, 15) which showed significantly higher rates in the ER group compared to the SH group (7.97% vs. 1.29%; *OR*=3.10, 95%*CI*: 1.21-7.97; *P*=0.02). Slight heterogeneity was observed *(I*² =23%), and sensitivity analysis showed that after excluding the studies of Ou et al. (4) and Buczkowski et al. (15), there was no significant difference between the mortality rates of the ER and SH groups (4.76% versus 1.81%; *OR*=1.28, 95% *CI*: 0.40-4.12, *P*=0.68, **Supplementary Figure 2F**). In addition, no heterogeneity was found among the studies (*I*²=0, *P*=0.49).

### 3.5 Postoperative tumor outcome measures

Four studies (2–4, 15) reported tumor recurrence, and *χ* ^2^ test suggested no heterogeneity (*I* ^2 =^ 0) among the studies. The fixed-effects model showed that the difference in total recurrence rate between the two groups was not statistically significant (69.3% vs. 64.3%); *OR*=1.11, 95%*CI*: 0.64-1.93, *P*=0.71, **Supplementary Figure 3A**). Four articles (2–4, 11) reported peritoneal metastases or recurrence, and showed no heterogeneity (*I^2 =^* 0). Fixed-effects model showed that the recurrence rate of peritoneal metastases was lower in the ER group, albeit not significantly (15.6% versus 17.1%; *OR*=0.80, 95%*CI*: 0.43-1.5, *P*=0.49, **Supplementary Figure 3B**).

### 3.6 Survival outcomes

All 8 included studies reported 1- and 2-year OS rates; 6 studies (2–4, 11, 12, 14) reported 3-year survival rates; and only 5 studies (2, 4, 11, 12, 14) had the 5-year OS data. The 1-, 2-, and 3-year OS rates were not significantly different between the ER and SH groups (*P*>0.05), and the respective hazard ratios (*HR*) were 1.06 (95% *CI*: 0.62-1.81, **Supplementary Figure 4A**), 1.38 (95% *CI*: 0.94-2.03, **Supplementary Figure 4B**) and 1.05 (95% *CI*: 0.64-1.72, **Supplementary Figure 4C**). The *χ* ^2^ test showed lack of heterogeneity between the studies (1 year OS: *I*²=0, *P*=0.84; 2 years OS: *I*²=0, *P*= 0.10; 3 years OS: *I*²=38%, *P*=0.84), and sensitivity analysis did not alter the statistical results. However, the 5-year OS of the ER group was significantly lower than that of the SH group (*HR* =1.52, 95% *CI*: 1.14-2.03, *P*=0.005, **Supplementary Figure 4D**), and there was no heterogeneity between the studies (*I* ²=0, *P*=0. 92).

Four studies (2–4, 11) reported the 1-, 2- and 3-year DFS, and only 3 studies (2, 4, 11) reported the DFS for 5 years. The 1-, 2-, 3- and 5-year DFS rates were similar in the ER and SH groups (*P*>0.05), with respective *HR* of 1.21 (95% *CI*: 0.78-1.87, **Supplementary Figure 5A**), 1.19 (95% *CI*: 0.86-1.65, **Supplementary Figure 5B**), 1.2 (95%*CI*: 0.88-1.63, **Supplementary Figure 5C**) and 1.27 (95%*CI*: 0.96-1.69, **Supplementary Figure 5D**). There was no heterogeneity between the studies (1 year DFS: *I*² =0, *P*=0. 68; 2 years DFS: *I*²=0, *P*=0.85; 3 years DFS: *I*²=0, *P*=0.99; 5 DFS: *I*²=0, *P*=0.45).

### 3.7 Sensitivity analysis and publication bias

Sensitivity analysis included the studies with mNOS scores of 7 and above. There was no change in the statistical results of the recent postoperative outcome measures and survival data. After two studies, the case fatality rate was not SH group due to the removal of Ou et al. (4) and Buczkowski et al. (15). Tested by Begg’s rank-related test (*P* =0.368, **Supplementary Figure 6A**) and Egger linear regression method (*P* =0.067, **Supplementary Figure 6B**) showed no publication bias in the studies included in this meta-analysis.

## 4 Discussion

### 4.1 Risk factors for rHCC

The current hypothesis is that the rapid expansion and invasion of the hepatic tumor leads to intra-plasmal hemorrhage of the tumor and obstructs the hepatic venous outflow tract, which causes intra-tumoral hypertension and eventual rupture (16–18). The risk factors of rHCC include cirrhosis, hypertension, tumors larger than 5 cm in diameter, thrombosis and extrahepatic infiltrates (16, 17, 19). Therefore, HCC patients with underlying diseases such as hypertension and cirrhosis, tumor > 5 cm in diameter, and extrahepatic infiltrates should be considered at high risk of tumor rupture, and radical resection should be performed at the earliest as long as the preoperative clinical evaluation is consistent with the surgical requirements.

### 4.2 Short-term survival of rHCC

A systematic review (20) pooled clinical data of 4941 patients with rHCC from 67 studies in a systematic review, and found that the average 30-day and 6-month survival rates were 66.9% and 53.6% respectively. The main causes of death were bleeding-related complications (34.3%) and liver failure (30.0%). In addition, the 30-day survival rate was 34.8% for the patients who received conservative medical care and did not undergo surgery or any other intervention, and 70.1% for patients who received transcatheter arterial chemoembolization (TACE) or TAE. Due to its minimal invasiveness, high selectivity, reproducibility, and low relative risk, TAE has a better hemostasis effect on patients with rHCC compared to simple open hemostasis, and can therefore prolong patient survival (2). Partial hepatectomy can remove ruptured tumors, clean the abdominal cavity, and achieve radical resection. Furthermore, compared to TAE or conservative medical treatment, radical resection is associated with lower mortality and better prognosis (21), and can improve the 30-day survival rate of rHCC patients to 95.5% or even 100% (20).

Due to the low incidence and heterogeneity of rHCC, the choice between ER or SH in patients with potentially resectable spontaneous rHCC is controversial. Zheng YJ et al. (22) conducted a meta-analysis of 7 retrospective studies comparing the outcomes of early hepatectomy (EH) or delayed hepatectomy (DH) on 385 patients with spontaneous rHCC, and found that DH (7 days after rupture) can reduce intraoperative bleeding, intraoperative blood transfusion, and 30-day mortality rate, and improve the 1-year, 2-year, and 3-year OS rate. There was no difference between the 5-year OS of the two groups. However, Zheng YJ et al. (22) defined EH as that performed within 3 days after the rupture of HCC, and DH as resection after 7 days of conservative treatment and/or scavenged hemostasis. However, the definition of operation time was vague, which could not fully meet the inclusion criteria of the meta-analysis, resulting in obvious selection bias. In addition, there was systematic error in extracting information from literature, the *HR* for 1-, 2-, 3-, and 5-year OS of rHCC patients reported by Zhong et al. (12) was 1.42 (95% *CI*: 0.35 to 5.82), and that reported by Buczkowski et al. (15) for 1-, 2- and 3-year OS was 3.74 (95%CI: 0.55 to 25.55). In addition, two studies (3, 11) published in 2019 and 2020 were not included in the meta-analysis.

### 4.3 Long-term survival of rHCC

Moris D et al. (20) summarized the long-term prognosis of patients with rHCC from 67 reports, and concluded that tumor recurrence and metastasis were the most frequent cause of death (17.2% of the overall cohort). As expected, surgical resection led to more favorable long-term outcomes. The 1-, 3- and 5-year OS in the ER group were 40%-94.6%, 41.1%-49.5%, and 23.3%-27.8% respectively, compared to 57.1%-90%, 19%-67.5%, and 7.6%-67.5% in the SH group.

The current meta-analysis showed the 1-, 2- and 3-year OS and the 1-, 2-, 3- and 5-year DFS rates were similar in ER and SH groups (all *P*>0.05), whereas the 5-year OS rate was significantly lower in the ER group (*HR*=1.52; 95%*CI*: 1.14-2.03, *P*=0.005). Although some studies have reached conclusions consistent with these results, they are limited by the small sample size and insufficient follow-up duration (2). One possible reason of the comparable 3-year survival rates of ER and SH is that the amount of intraperitoneal hemorrhage is counted in the ER group, and the time from TAE to resection varies from 1 day to 2 months. The hemorrhage partially absorbed and removed in the SH group, which may explain similar survival prognosis of both groups within 3 years. However, the 5-year OS in the ER group was significantly shorter than that in the SH group. It is difficult at present to provide a convincing explanation for this difference, which may not be due to the treatment at the time of HCC rupture but rather due to the follow-up treatment measures after radical resection. This hypothesis will have to be validated with larger samples and longer follow-up evaluation. Therefore, based on the aggregated data, rHCC should not be considered a “single clinical event” and “rupture” should not be considered as the only adverse prognostic factor.

### 4.4 Survival prediction of rHCC

Given the paucity of studies on spontaneously rHCC after radical resection, and the significant heterogeneity between cases with non-ruptured and rHCC, it is still unclear whether liver tumor rupture affects long-term survival. In addition, the survival benefits of the different treatment methods are not consistent. Therefore, it is essential to identify novel prognostic markers or models for rHCC in order to aid clinical decision-making (**Table 1**).

**Table 1:** The models of predicting the prognosis of rHCC.

	Model	Author	Sample/Method	Risk factor		Outcome
TAE for rHCC	Prediction of prognosis of TAE treatment of rHCC by imaging and clinical scoring systems	Ngan H et al	33/Mantel-Cox test	Total bilirubin=2.9 mg/dl		mOS 1 week
				Total bilirubin<2.9 mg/dl		mOS 15 weeks
		Okazaki M et al	38/Mantel-Cox test	Total bilirubin=3.0 mg/dl		mOS 13 days
				Total bilirubin ≤3.0 mg/dl		mOS 165 days
		Lee KH et al	111/Multiple logistic regression model	Bilobar tumor distribution(3points)	High risk≥4points	30 days mortality 86.8%
				Total bilirubin=2.5mg/dL(2points)	Moderate risk=3points	30 days mortality 31.8%
				Albumin <30g/L(1points)	Low risk ≤ 2points	30 days mortality 2.6%
		Fan WZ et al	94/Cox regression analysis	Shock index	≥0.6=<1	mOS 12.0 ± 1.0 days
					≥1	mOS 52.0 ± 7.2 days
				Child-Pugh score	10/11	mOS 51.0 ± 13.9 days
					12/13	mOS 28.0 ± 3.7 days
				Portal vein tumor thrombus	Main	mOS 14.0 ± 2.0 days
					Lobar	mOS 34.0 ± 5.1 days
					Segmental	mOS 52.0 ± 6.9 days
	MELD predicts TAE for rHCC	Jundt MC et al	24/Log-rank test	MELD-Na score=16		mOS 9 days, 30 days mortality 67%
				MELD-Na score ≤ 16		mOS 166.5 days, 30 days mortality 21%
Partial liver resection for rHCC	TAA	Wu JJ et al	139/Log-rank test	Scores according to the tumor size	High risk 10-13 points	1 year OS 30.2%
				Scores according to the AFP	Moderate risk 6-9 points	1 year OS 43.2%
				Scores according to the ALP	Low risk 0-5 points	1 year OS 88.1%
	AFP	Chua DW et al	79/Cox regression analysis	AFP=200 ng/mL		1 year OS 33.3%
				Tumor size=10 cm		1year recurrent rate 90.9%
		She WH et al.	114/Log-rank test	AFP≥256 ng/mL		mDFS 5.9 months
				AFP<256 ng/ml		mDFS 10.7 months

TAE, transcatheter artery embolization; rHCC, ruptured hepatocellular carcinoma; MELD, Model for End-Stage Liver Disease; mOS, median overall survival; TAA, tumor-associated antigen; AFP, alpha-fetoprotein; mDFS, median disease-free survival.

#### 4.4.1 TAE for rHCC

Since HCC rupture causes acute bleeding, the primary goal of treatment is to stem the bleeding and prevent internal hemorrhage. TAE is a minimally invasive and reproducible approach with a good hemostasis effect in patients with hepatic tumor rupture. However, it is not suitable for all patients with rHCC.

##### 4.4.1.1 Prediction of prognosis of TAE treatment of rHCC by imaging and clinical scoring systems

Compared to single abdominal hemostasis, emergency TAE has a better hemostasis effect on patients with rHCC, and can prolong patient survival (2). However, Ngan H et al. (23) reported that emergency TAE provided little survival benefit to patients with total bilirubin levels > 2.92 mg/dL, and Okazaki et al. (24) considered total bilirubin level > 3 mg/dL to be a contraindication to TAE. Lee et al. (25) devised a scoring system by combining imaging and clinical laboratory parameters to predict the case fatality rate in patients with rHCC at 30 days after TAE, and identified bilobar tumors, total bilirubin > 2.5 mg/dL and albumin < 30 g/L as independent predictors of 30-day fatality. Patients with rHCC have poor liver function, and underlying cirrhosis and liver dysfunction in most cases, which respond poorly to conservative treatment alone. Fan WZ et al. (26) consider emergency TAE to be an effective intervention in patients with Child-Pugh C grade rHCC with hepatic shock, particularly in those with shock index ≥1, Child-Pugh score 10/11 and grade 1 or lower branch portal vein cancer suppository. In contrast, the efficacy of TAE and conservative medical treatment were similar in patients with Child-Pugh score 12/13 tumors and portal vein trunk carcinoma suppositories.

##### 4.4.1.2 Model for End-Stage Liver Disease (MELD) predicts prognosis for TAE treatment of rHCC

MELD scores are based on total serum bilirubin concentration, international normalized ratio of prothrombin time, and serum creatinine concentration (27). In addition, serum sodium concentration has been recognized as an important prognostic factor in patients with cirrhosis, and hyponatremia is associated with ascites (28), hepatorenal syndrome (29), and liver disease death (30). The combination of MELD score and serum sodium concentration (MELD-Na) can predict the case fatality rate of liver transplants with greater accuracy. Jundt MC et al. (31) used the MELD-Na score to evaluate the perioperative and short-term case fatality rates of rHCC patients undergoing TAE, and found that MELD-Na was an independent risk factor of post-TAE survival. Higher MELD-Na scores were associated with worse baseline liver function and tumor prognosis, and the 30-day and 90-day case fatality rates of patients with MELD-Na score >16 were respectively 67% and 89% after TAE. Thus, rHCC patients with a Child-Pugh score >11, MELD-Na score >16 and portal vein main trunk carcinoma suppositories have extremely poor short- and long-term prognosis, and emergency intervention does not improve their chances of survival compared to conservative treatment.

#### 4.4.2 Partial liver resection for rHCC

Most rHCC patients have underlying cirrhosis and decompensated liver function, which can be aggravated due to surgery. Furthermore, surgery also increases the risk of jaundice and refractory ascites, eventually leading to liver and kidney failure. Therefore, it is crucial to screen for the suitable patients.

##### 4.4.2.1 Predictive model for partial hepatic resection of rHCC

Wu JJ et al. (32) conducted an univariate and multivariate analysis of 139 patients with rHCC, and established a new tumor-associated antigen (TAA) scoring model based on tumor diameter, alkaline phosphatase (ALP), and alpha-fetoprotein (AFP). Approximately 88.1% of the low-risk patients survived for more than 1 year compared to only 43.2% and 30.2% of the intermediate-risk and high-risk patients respectively (P<0.001). The 2-, 3-, and 5-year OS rates were 73.8%, 64.1%, and 44.2% respectively in the low-risk group, 27.3%, 24.8%, and 15.5% in the moderate-risk group, and 9.3%, 4.7%, and 0 in the high-risk group. The DFS rates also showed significant differences with the new staging model (32).

Compared to the Barcelona Clinic Liver Cancer (BCLC) and the Cancer of the Liver Italian Program (CLIP) classification models, the TAA model showed a higher Harrell’s C statistic, indicating greater predictive accuracy for the postoperative prognosis of rHCC. In addition, the TAA model has lower Akaike Information Criterion (AIC) compared to BCLC and CLIP, indicating that the model fits well and loses less information when predicting OS (relative probability <0.001). Thus, the TAA model has better discrimination power and homogeneity than the BCLC and CLIP systems for predicting the OS and DFS of rHCC patients after surgical resection (32).

##### 4.4.2.2 AFP predicts prognosis after rHCC resection

AFP is an important diagnostic and prognostic marker of HCC, and studies increasingly show that elevated AFP is associated with increased tumor burden (33, 34) and poor prognosis (21). However, it is unclear whether AFP levels can predict the survival in patients with rHCC. AFP > 200 ng/mL (35) or >1000 ng/ml (21) have been identified as independent risk factors for the overall survival of rHCC patients. In addition, tumor size > 10 cm and AFP > 200 ng/mL are associated with early postoperative recurrence rates of 54.5%-90.9% and perioperative case fatality rate of 66.7% in patients with rHCC, and are thus useful indicators for avoiding futile surgery (36). She WH et al. (37) showed that AFP ≥ 256 ng/mL is an independent risk factor of OS in rHCC patients, and portends worse survival regardless of tumor size. Thus, surgical intervention (ER or SH) is recommended for rHCC patients with low TAA score and AFP < 256 ng/mL. Although surgical resection is still the first choice for increasing the chances of survival in patients with higher TAA scores (6 to 9) and AFP ≥ 256 ng/mL, postoperative adjuvant therapy should be considered for lowering the risk of tumor recurrence.

### 4.5 Treatment of rHCC survivors

Adjuvant treatment after curative hepatectomy is a crucial factor influencing patient survival. However, data regarding the safety and efficacy of sorafenib in rHCC patients is limited. One single-center study showed that the cumulative survival rates in an rHCC cohort (38) after 4, 8, and 12 months of surgery were higher for the patients that received sorafenib as an adjuvant treatment. Postoperative TACE can also be used as adjuvant therapy to prevent recurrence after hepatectomy (39), although perioperative TACE decreases intrahepatic metastasis but increases peritoneal dissemination in rHCC patients. Recently, Huang A et al. (40) found that adjuvant TACE conferred a survival benefit in patients with a high risk of recurrence (multiple tumors, as well as micro- and macro-vascular invasion). However, these results should be interpreted with caution since their sample size was limited (38–41). Few studies have focused on the treatment of rHCC survivors, and the strategies are mainly determined based on the tumor burden after recurrence. Targeted therapies and immunotherapy are increasingly being considered for the management of advanced HCC (42).

In 2014, Zheng SZ et al. (38) conducted a retrospective study on a cohort of 32 rHCC patients to determine the efficacy and safety of sorafenib. Twenty-two patients in the cohort had undergone surgery (ER or SH), 10 received TAE or TACE, and 12 received sorafenib postoperatively. The initial dose of sorafenib was 200 mg bid, and increased to the full dose of 400 mg bid after 5 to 7 days in case there was no toxicity. The median survival duration of the surgery group (n=12) was 11.41 months, and that of the surgery + sorafenib group (n= 10) was 16.47 months. In contrast, the median survival duration in the surgery/TAE/TACE group (n=20) was only 8.32 months, compared to 16.41 months in the surgery/TAE/TACE+sorafenib group (n=12) (P=0.04). In addition, 2 patients achieved complete radiological remission, 3 patients were stable, and 7 patients developed tumors. Three patients were temporarily administered with a reduced dose of sorafenib due to toxicity, and the main side effects were hand-foot skin reactions and diarrhea rather than any serious adverse reactions.

Thus, survivors of radical surgical resection (ER or SH) can be treated with adjuvant TACE, targeted drugs, immune checkpoint inhibitors, or hepatic artery perfusion chemotherapy based on locally advanced or advanced HCC.

## 5 Summary

It is often difficult to stratify rHCC patients based on clinical presentation and biochemical data to determine appropriate treatment strategies. There was no significant difference in the short-term efficacy of ER or SH in the treatment of ruptured HCC, and SH was superior to ER in the long-term survival. Identification of novel prognostic indicators or models of rHCC may help guide treatment decisions and improve outcomes.

## Data availability statement

The original contributions presented in the study are included in the article/**Supplementary Material**. Further inquiries can be directed to the corresponding authors.

## Author contributions

All authors contributed to the study concept, design, data interpretation, and discussion. YR and SY contributed to the screening and data collection. XB and DL contributed to the assessment of the included article. ZH and XL contributed to the data analysis. XH and CW contributed to the writing of the manuscript. JZ and XC contributed to provide expert insight into the revision of the manuscript and being as corresponding author. All authors approved the final version of the reports.

## Funding

This research was supported by Sanming Project of Medicine in Shenzhen (No.SZSM202011010) and Shenzhen High-level Hospital Construction Fund.

## Acknowledgments

The authors acknowledge Zhen Huang, MD, Ph.D., Hong Zhao, MD, Ph.D. work in National Cancer Center/Cancer Hospital, Chinese Academy of Medical Sciences and Peking Union Medical College, for the writing assistance and critical revision of the manuscript for important intellectual content.

## Conflict of interest

The authors declare that the research was conducted in the absence of any commercial or financial relationships that could be construed as a potential conflict of interest.

## Publisher’s note

All claims expressed in this article are solely those of the authors and do not necessarily represent those of their affiliated organizations, or those of the publisher, the editors and the reviewers. Any product that may be evaluated in this article, or claim that may be made by its manufacturer, is not guaranteed or endorsed by the publisher.

## References

[1] Forner A Reig M Bruix J . Hepatocellular carcinoma. Lancet (London England) (2018) 391(10127):1301–14. doi: 10.1016/s0140-6736(18)30010-2 29307467

[2] Ren A Luo S Ji L Yi X Liang J Wang J . Peritoneal metastasis after emergency hepatectomy and delayed hepatectomy for spontaneous rupture of hepatocellular carcinoma. Asian J Surg (2019) 42(2):464–9. doi: 10.1016/j.asjsur.2018.09.006 30420157

[3] Zhou C Zhang C Zu QQ Wang B Zhou CG Shi HB . Emergency transarterial embolization followed by staged hepatectomy versus emergency hepatectomy for ruptured hepatocellular carcinoma: A single-center, propensity score matched analysis. Jpn J Radiol (2020) 38(11):1090–8. doi: 10.1007/s11604-020-01007-2 32564291

[4] Ou D Yang H Zeng Z Luo Y Yang L . Comparison of the prognostic influence of emergency hepatectomy and staged hepatectomy in patients with ruptured hepatocellular carcinoma. Dig Liver Dis (2016) 48(8):934–9. doi: 10.1016/j.dld.2016.04.016 27263055

[5] Subramaniam S Kelley RK Venook AP . A review of hepatocellular carcinoma (HCC) staging systems. Chin Clin Oncol (2013) 2(4):33. doi: 10.3978/j.issn.2304-3865.2013.07.05 25841912

[6] Nuño-Guzmán CM Marín-Contreras ME . Ruptured hepatocellular carcinoma and non-alcoholic fatty liver disease, a potentially life-threatening complication in a population at increased risk. Ann Hepatol (2020) 19(1):3–4. doi: 10.1016/j.aohep.2019.11.001 31916949

[7] Aoki T Kokudo N Matsuyama Y Izumi N Ichida T Kudo M . Prognostic impact of spontaneous tumor rupture in patients with hepatocellular carcinoma: An analysis of 1160 cases from a nationwide survey. Ann Surg (2014) 259(3):532–42. doi: 10.1097/SLA.0b013e31828846de 23478524

[8] Hiraoka A Kawamura T Aibiki T Okudaira T Toshimori A Yamago H . Prognosis and therapy for ruptured hepatocellular carcinoma: Problems with staging and treatment strategy. Eur J Radiol (2015) 84(3):366–71. doi: 10.1016/j.ejrad.2014.11.038 25554005

[9] Chan AC Dai JW Chok KS Cheung TT Lo CM . Prognostic influence of spontaneous tumor rupture on hepatocellular carcinoma after interval hepatectomy. Surgery (2016) 159(2):409–17. doi: 10.1016/j.surg.2015.07.020 26294087

[10] Moher D Liberati A Tetzlaff J Altman DG . Preferred reporting items for systematic reviews and meta-analyses: the PRISMA statement. BMJ (2009) 339:b2535. doi: 10.1136/bmj.b2535 19622551 PMC2714657

[11] Wu JJ Zhu P Zhang ZG Zhang BX Shu C Mba'nbo-Koumpa AA . Spontaneous rupture of hepatocellular carcinoma: Optimal timing of partial hepatectomy. Eur J Surg Oncol (2019) 45(10):1887–94. doi: 10.1016/j.ejso.2019.02.033 31405632

[12] Zhong F Cheng XS He K Sun SB Zhou J Chen HM . Treatment outcomes of spontaneous rupture of hepatocellular carcinoma with hemorrhagic shock: A multicenter study. SpringerPlus (2016) 5(1):1101. doi: 10.1186/s40064-016-2762-8 27468402 PMC4947465

[13] Yang H Chen K Wei Y Liu F Li H Zhou Z . Treatment of spontaneous ruptured hepatocellular carcinoma: A single-center study. Pak J Med Sci (2014) 30(3):472–6. doi: 10.12669/pjms.303.4001 PMC4048488 24948961

[14] Hsueh KC Fan HL Chen TW Chan DC Yu JC Tsou SS . Management of spontaneously ruptured hepatocellular carcinoma and hemoperitoneum manifested as acute abdomen in the emergency room. World J Surg (2012) 36(11):2670–6. doi: 10.1007/s00268-012-1734-6 22864567

[15] Buczkowski AK Kim PT Ho SG Schaeffer DF Lee SI Owen DA . Multidisciplinary management of ruptured hepatocellular carcinoma. J Gastrointest Surg (2006) 10(3):379–86. doi: 10.1016/j.gassur.2005.10.012 16504883

[16] Zhu LX Wang GS Fan ST . Spontaneous rupture of hepatocellular carcinoma. Br J Surg (1996) 83(5):602–7. doi: 10.1002/bjs.1800830507 8689200

[17] Rossetto A R Adani GL Risaliti A Baccarani U Bresadola V Lorenzin D . Combined approach for spontaneous rupture of hepatocellular carcinoma. World J Hepatol (2010) 2(1):49–51. doi: 10.4254/wjh.v2.i1.49 21160956 PMC2999262

[18] Xia F Ndhlovu E Zhang M Chen X Zhang B Zhu P . Ruptured hepatocellular carcinoma: Current status of research. Front Oncol (2022) 12:848903. doi: 10.3389/fonc.2022.848903 35252016 PMC8891602

[19] Zhu Q Li J Yan JJ Huang L Wu MC Yan YQ . Predictors and clinical outcomes for spontaneous rupture of hepatocellular carcinoma. World J Gastroenterol (2012) 18(48):7302–7. doi: 10.3748/wjg.v18.i48.7302 PMC3544034 23326137

[20] Moris D Chakedis J Sun SH Spolverato G Tsilimigras DI Ntanasis-Stathopoulos I . Management, outcomes, and prognostic factors of ruptured hepatocellular carcinoma: A systematic review. J Surg Oncol (2018) 117(3):341–53. doi: 10.1002/jso.24869 29116644

[21] Zhang W Zhang ZW Zhang BX Huang ZY Zhang WG Liang HF . Outcomes and prognostic factors of spontaneously ruptured hepatocellular carcinoma. J Gastrointest Surg (2019) 23(9):1788–800. doi: 10.1007/s11605-018-3930-7 30328072

[22] Zheng YJ Z Li DL Luo D Chen XP Zhang B Fang C . Early versus delayed hepatectomy for spontaneously ruptured hepatocellular carcinoma: A systematic review and meta-analysis. J Invest Surg (2021) 34(11):1214–22. doi: 10.1080/08941939.2020.1792009 32654535

[23] Ngan H Tso WK Lai CL Fan ST . The role of hepatic arterial embolization in the treatment of spontaneous rupture of hepatocellular carcinoma. Clin Radiol (1998) 53(5):338–41. doi: 10.1016/s0009-9260(98)80004-4 9630270

[24] Okazaki M Higashihara H Koganemaru F Nakamura T Kitsuki H Hoashi T . Intraperitoneal hemorrhage from hepatocellular carcinoma: emergency chemoembolization or embolization. Radiology (1991) 180(3):647–51. doi: 10.1148/radiology.180.3.1651524 1651524

[25] Lee KH Tse MD Law M Cheng AK Wong HF Yu ML . Development and validation of an imaging and clinical scoring system to predict early mortality in spontaneous ruptured hepatocellular carcinoma treated with transarterial embolization. Abdom Radiol (NY) (2019) 44(3):903–11. doi: 10.1007/s00261-019-01895-7 30631903

[26] Fan WZ Zhang YQ Yao W Wang Y Tan GS Huang YH . Is emergency transcatheter hepatic arterial embolization suitable for spontaneously ruptured hepatocellular carcinoma in child-pugh c cirrhosis? J Vasc Interv Radiol (2018) 29(3):404–12.e3. doi: 10.1016/j.jvir.2017.09.022 29249595

[27] Kamath PS Wiesner RH Malinchoc M Kremers W Therneau TM Kosberg CL . A model to predict survival in patients with end-stage liver disease. Hepatology (2001) 33(2):464–70. doi: 10.1053/jhep.2001.22172 11172350

[28] Arroyo V Colmenero J . Ascites and hepatorenal syndrome in cirrhosis: Pathophysiological basis of therapy and current management. J Hepatol (2003) 38:69–89. doi: 10.1016/S0168-8278(03)00007-2 12591187

[29] Porcel A Díaz F Rendón P Macías M Martín-Herrera L Girón-González JA . Dilutional hyponatremia in patients with cirrhosis and ascites. Arch Intern Med (2002) 162(3):323–8. doi: 10.1001/archinte.162.3.323 11822925

[30] Fernández-Esparrach G Sánchez-Fueyo A Ginès P Uriz J Quintó L Ventura P-J . A prognostic model for predicting survival in cirrhosis with ascites. J Hepatol (2001) 34(1):46–52. doi: 10.1016/S0168-8278(00)00011-8 11211907

[31] Jundt MC Owen RL Thompson SM Fleming CJ Stockland AH Andrews JC . MELD-Na > 16 is associated with high peri-procedural and short-term mortality in patients with ruptured hepatocellular carcinoma treated with emergent transarterial embolization. Abdom Radiol (NY) (2022) 47(1):416–22. doi: 10.1007/s00261-021-03306-2 34633495

[32] Wu J Zhu P Zhang Z Zhang B Shu C Chen L . A new tumor-associated antigen prognostic scoring system for spontaneous ruptured hepatocellular carcinoma after partial hepatectomy. Cancer Biol Med (2018) 15(4):415–24. doi: 10.20892/j.issn.2095-3941.2018.0095 PMC6372911 30766751

[33] She WH Chan ACY Cheung TT Lo CM Chok KSH . Survival outcomes of liver transplantation for hepatocellular carcinoma in patients with normal, high and very high preoperative alpha-fetoprotein levels. World J Hepatol (2018) 10(2):308–18. doi: 10.4254/wjh.v10.i2.308 PMC5838449 29527266

[34] Hameed B Mehta N Sapisochin G Roberts JP Yao FY . Alpha-fetoprotein level > 1000 ng/mL as an exclusion criterion for liver transplantation in patients with hepatocellular carcinoma meeting the Milan criteria. Liver Transpl (2014) 20(8):945–51. doi: 10.1002/lt.23904 PMC4807739 24797281

[35] Kerdsuknirun J Vilaichone V Vilaichone RK . Risk factors and prognosis of spontaneously ruptured hepatocellular carcinoma in Thailand. Asian Pac J Cancer Prev (2018) 19(12):3629–34. doi: 10.31557/apjcp.2018.19.12.3629 PMC6428545 30583692

[36] Chua DW Koh YX Liew YX Chan CY Lee SY Cheow PC . Pre-operative predictors of early recurrence/mortality including the role of inflammatory indices in patients undergoing partial hepatectomy for spontaneously ruptured hepatocellular carcinoma. J Surg Oncol (2018) 118(8):1227–36. doi: 10.1002/jso.25281 30399204

[37] She WH Chan MY Ma KW Tsang SHY Dai WC Chan ACY . Alpha-fetoprotein in predicting survival of patients with ruptured hepatocellular carcinoma after resection. J Invest Surg (2022) 35(5):1–7. doi: 10.1080/08941939.2021.2012615 34865574

[38] Zheng SZ Liu DJ Sun P Yu GS Xu YT Gong W . Feasibility and safety of sorafenib treatment in hepatocellular carcinoma patients with spontaneous rupture. World J Gastroenterol (2014) 20(43):16275–81. doi: 10.3748/wjg.v20.i43.16275 PMC4239517 25473183

[39] Wang Z Ren Z Chen Y Hu J Yang G Yu L . Adjuvant transarterial chemoembolization for HBV-related hepatocellular carcinoma after resection: A randomized controlled study. Clin Cancer Res (2018) 24(9):2074–81. doi: 10.1158/1078-0432.ccr-17-2899 29420221

[40] Huang A Guo DZ Wang YP Fan J Yang XR Zhou J . The treatment strategy and outcome for spontaneously ruptured hepatocellular carcinoma: A single-center experience in 239 patients. J Cancer Res Clin Oncol (2022). doi: 10.1007/s00432-022-03916-3 PMC11800789 35118561

[41] Roussel E Bubenheim M Le Treut YP Laurent A Herrero A Muscari F . Peritoneal carcinomatosis risk and long-term survival following hepatectomy for spontaneous hepatocellular carcinoma rupture: Results of a multicenter French study (FRENCH-AFC). Ann Surg Oncol (2020) 27(9):3383–92. doi: 10.1245/s10434-020-08442-5 32285281

[42] Huang X Xu L Ma T Yin X Huang Z Ran Y . Lenvatinib plus immune checkpoint inhibitors improve survival in advanced hepatocellular carcinoma: A retrospective study. Front Oncol (2021) 11:751159. doi: 10.3389/fonc.2021.751159 34868952 PMC8637826

