# Supplementary figures and images for: Research progress of spontaneous ruptured hepatocellular carcinoma: Systematic review and meta-analysis
Source: Front Oncol. 2022 Sep 29;12:973857. doi: 10.3389/fonc.2022.973857 (PMC9559597; doi:10.3389/fonc.2022.973857)

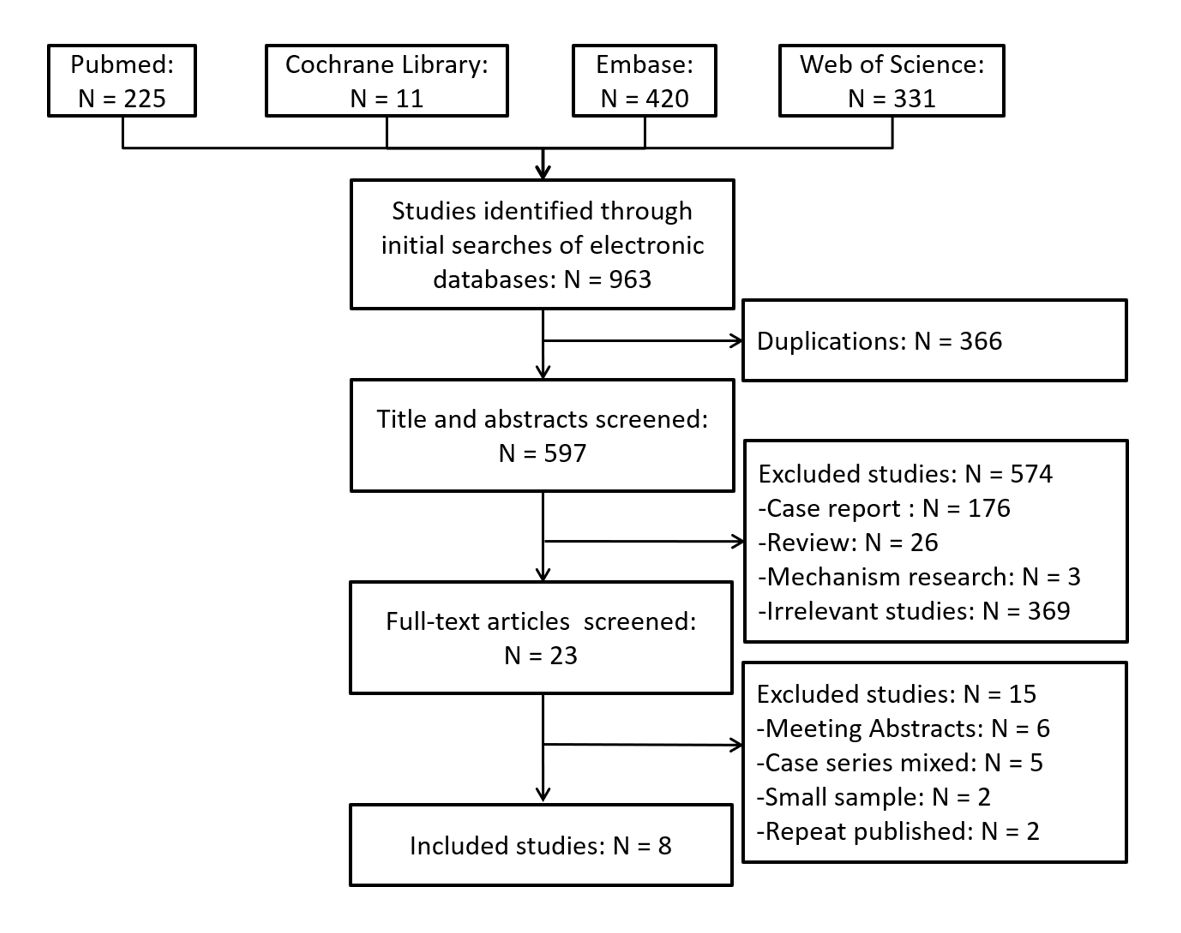

Supplement: Supplementary file 1 [file Image_1.tif]

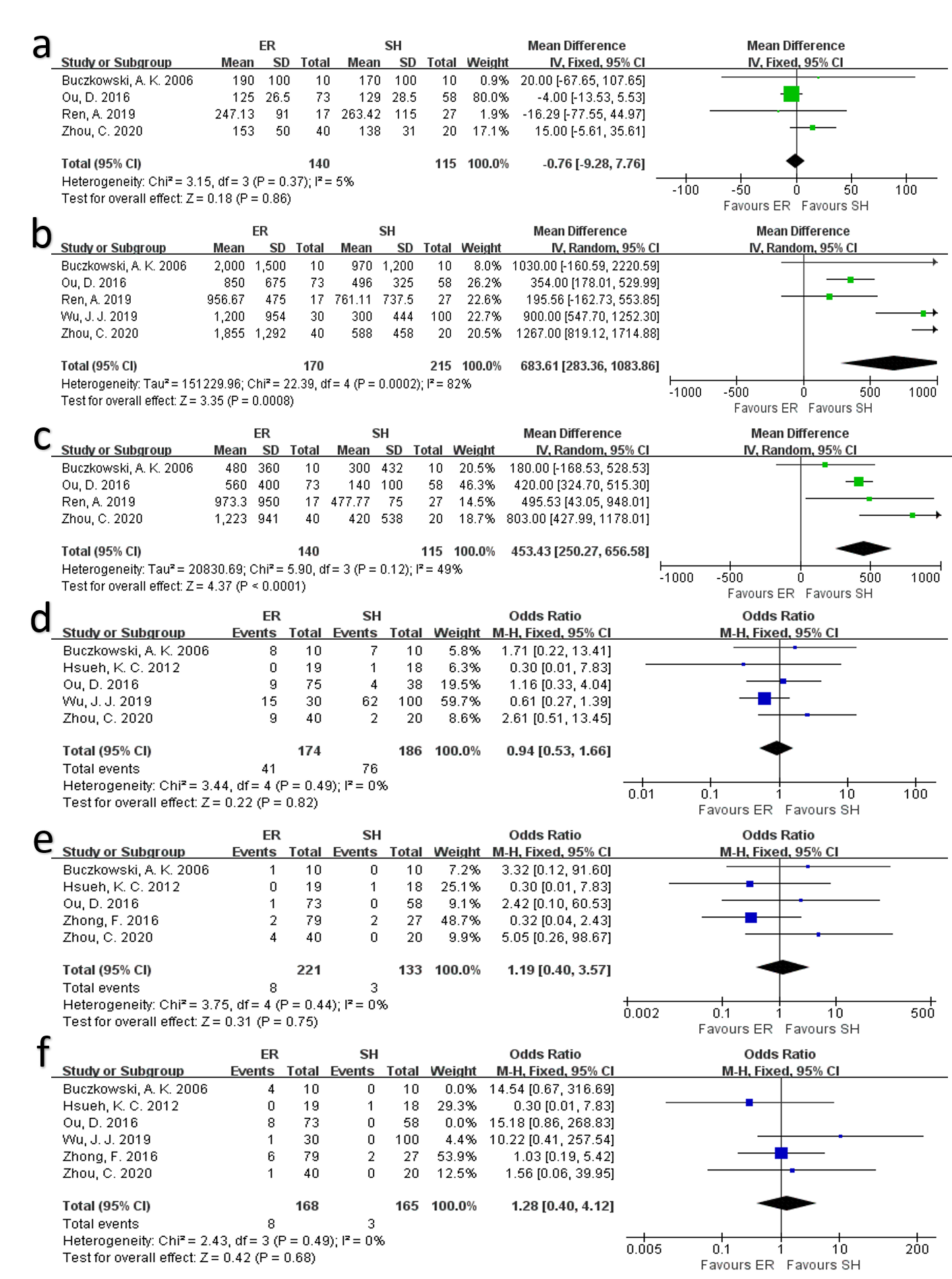

Supplement: Supplementary file 2 [file Image_2.png]

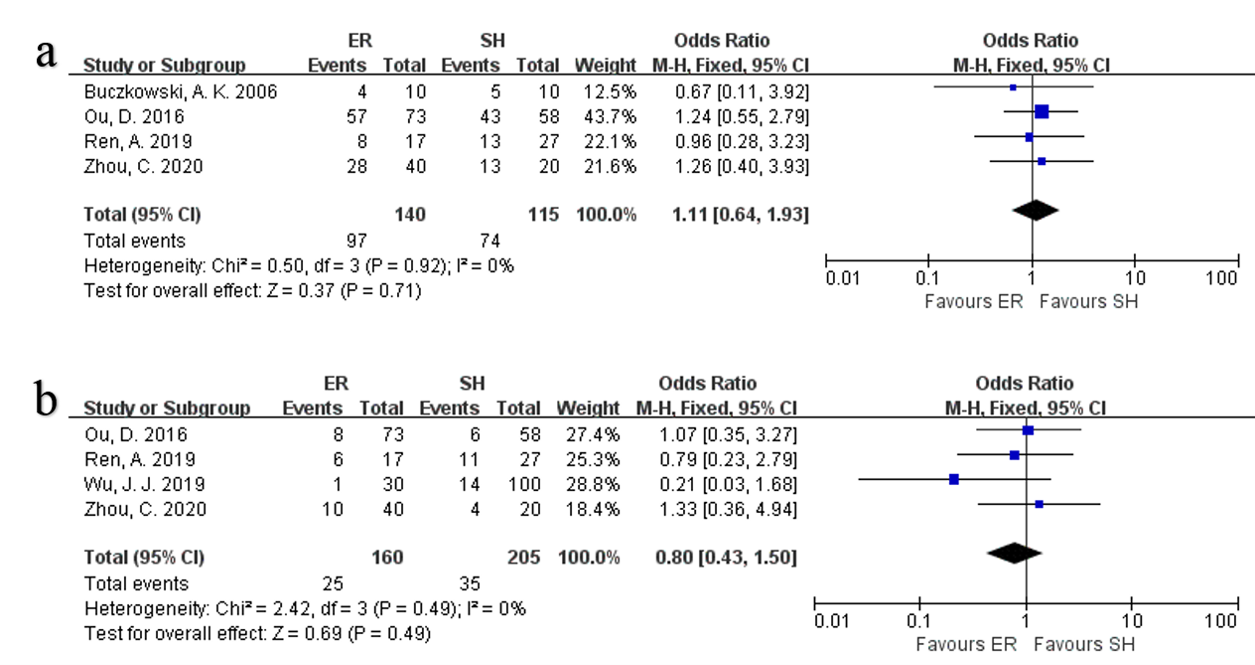

Supplement: Supplementary file 3 [file Image_3.png]

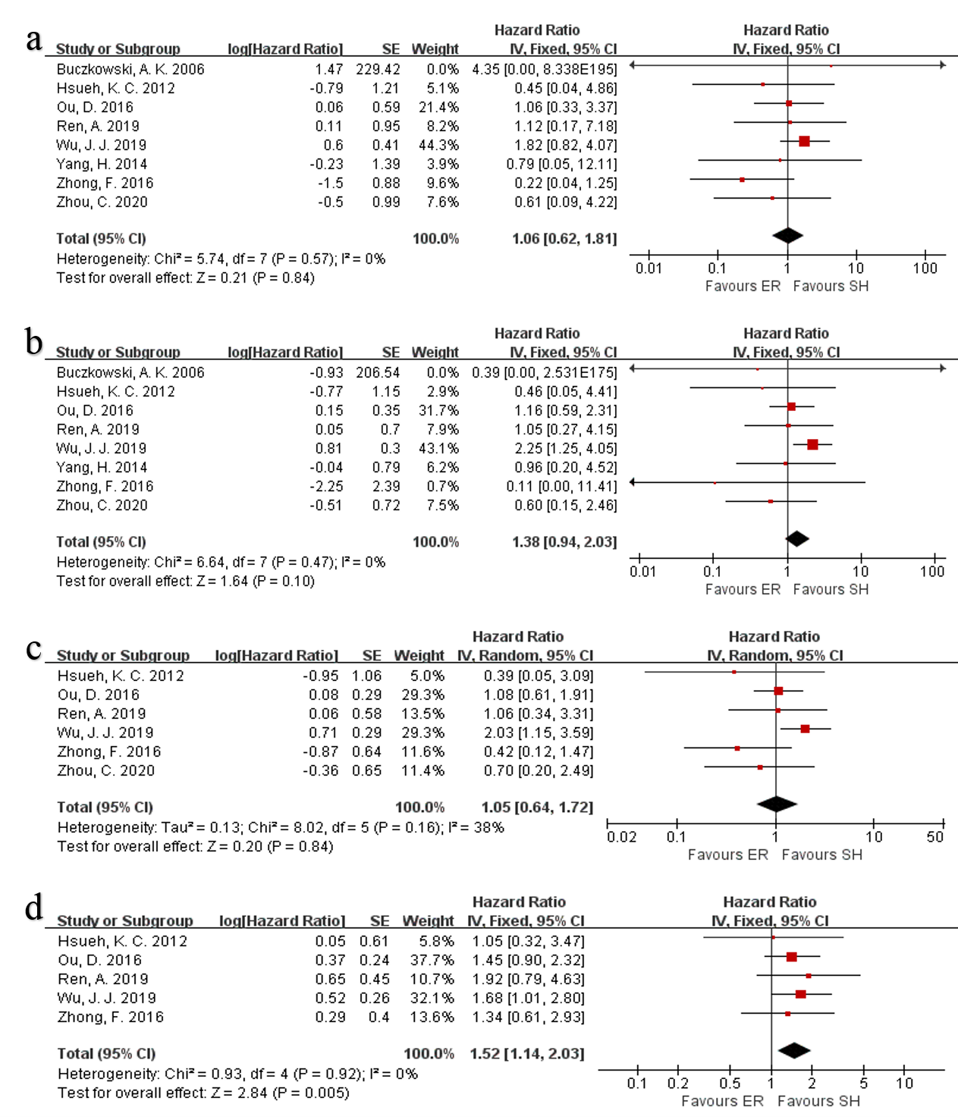

Supplement: Supplementary file 4 [file Image_4.png]

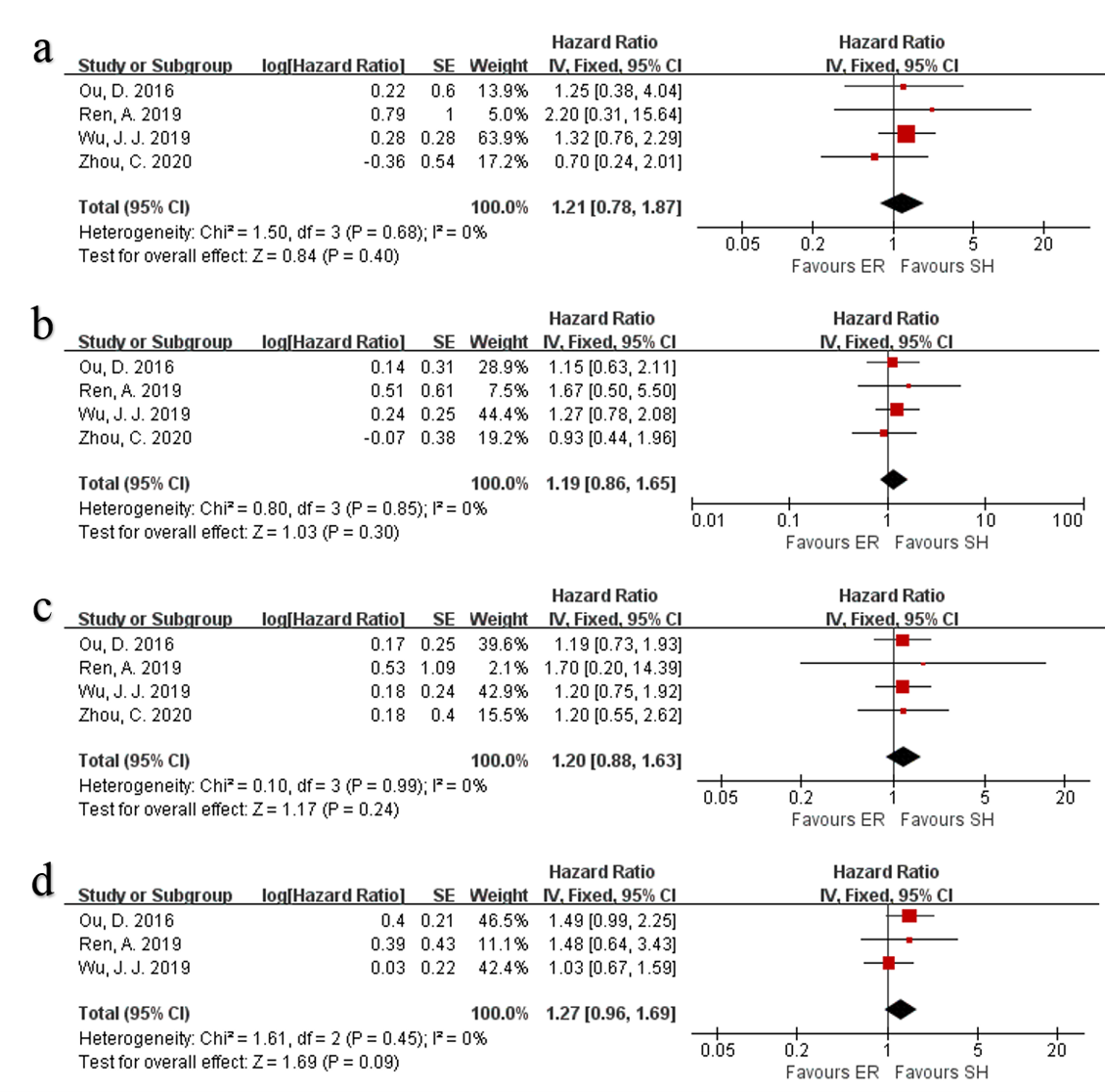

Supplement: Supplementary file 5 [file Image_5.png]

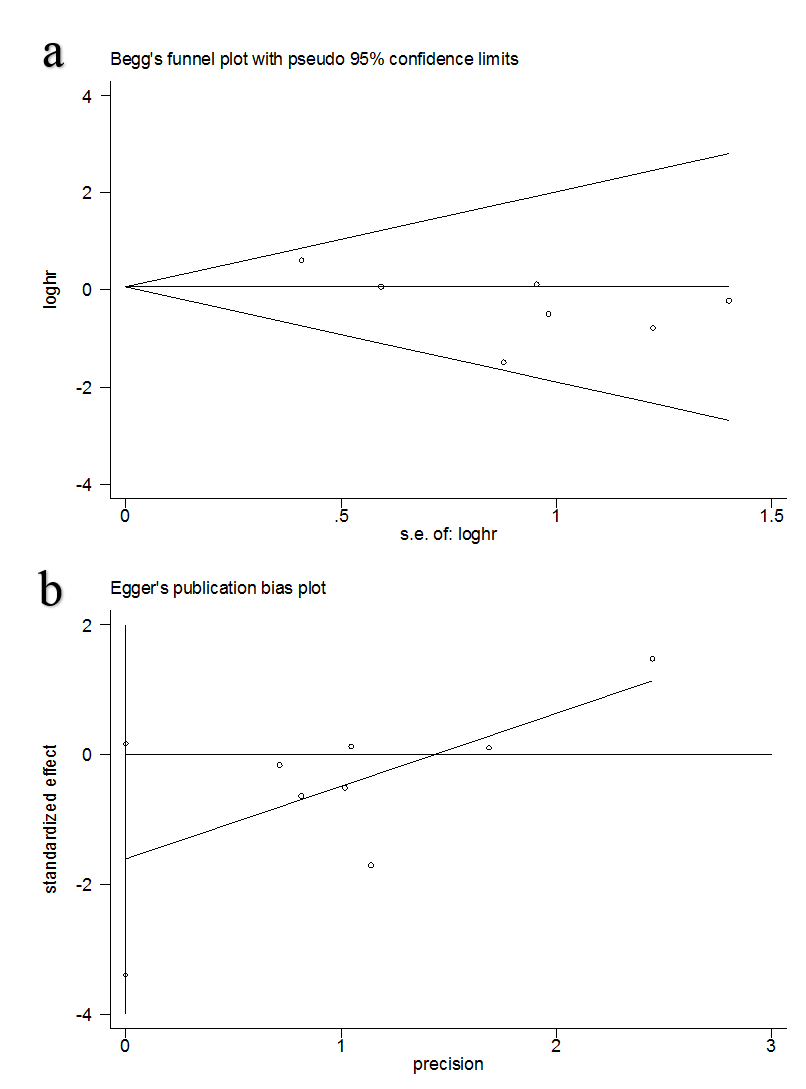

Supplement: Supplementary file 6 [file Image_6.png]
