# Supplementary material for: Research progress of spontaneous ruptured hepatocellular carcinoma: Systematic review and meta-analysis
Source: Front Oncol. 2022 Sep 29;12:973857. doi: 10.3389/fonc.2022.973857 (PMC9559597; doi:10.3389/fonc.2022.973857)
Supplement: Supplementary file 7 [file DataSheet_1.pdf]

Supplementary Material

Supplementary Figures

Supplementary Figure 1: Schematic illustration of the literature search and study-selection criteria.

Supplementary Figure 2: (a) Forest plot for operation time showed no significant difference between ER and SH. (b) Forest plot for blood loss showed that the ER group lost more blood than the SH group. (c) Forest plot for the need for blood transfusion was also significantly higher in the ER group. Forest plot for (d) the complication rates, (e) the postoperative liver failure and (f) the mortality rates of the ER and SH group were no significant difference.

Supplementary Figure 3: Forest plots for showed that the difference in (a) total recurrence rate and (b) peritoneal metastases between ER and SH group was not statistically significant.

Supplementary Figure 4: Forest plots for the (a) 1-, (b) 2-, and (c) 3-year OS rates were not significantly different between the ER and SH groups. (d) Forest plots for the 5-year OS of the ER group was significantly lower than that of the SH group.

Supplementary Figure 5: Forest plots for the (a) 1-, (b) 2-, (c) 3-year and (d) 5-year DFS rates were not significantly different between the ER and SH groups.

Supplementary Figure 6: Begg's test for does not indicate any evidence of publication bias.

Tested by (a) Begg's rank-related test (P =0.806) and (b) Egger linear regression method (P =0.067) showed no publication bias in the studies included in this meta-analysis.

Supplementary Tables

Supplementary Table 1 Summary of characteristics of included studies

Study	No. patients		Childs-Pugh A [n(%)]		Tumor size [cm,($\bar{x}$±s) or M(Q_1,Q_3)]		Shock [n(%)]		Liver cirrhosis [n(%)]	
	ER	SH	ER	SH	ER	SH	ER	SH	ER	SH
Zhou C	40	20	31(77.5)	11(55.0)	7.1±3.9	7.3±2.3	15(37.5)	10(50)	31(77.5)	15(75.0)
Wu JJ	30	100	17(56.7)	90(90.0)	6.5(4.8~8.5)	8.0(5.3~10)	15(50.0)	8(8.0)	15(50.0)	75(75.0)
Zhong F	79	27	53(67.1)	15(55.6)	8.8±2.3	9.0±1.4	NR	NR	66(83.5)	20(74.1)
Yang H	17	11	10(58.8)	8(72.7)	NR	NR	NR	NR	NR	NR
Ren A	17	27	NR	NR	8.6±3.4	10.4±4.9	NR	NR	8(47.1)	14(51.9)
Ou D	73	58	61(83.6)	47(81.0)	10(4~23)	11(6~25)	37(50.7)	25(43.1)	48(65.8)	37(63.8)
Hsueh KC	19	18	NR	NR	NR	NR	NR	NR	NR	NR
Buczkowski AK	10	10	NR	NR	7±4	7±3	NR	NR	7(70.0)	5(50.0)

ER: emergency resection; SH: staged hepatectomy; NR: no report

Supplementary Table 2 Risk of bias using the modified Newcastle-Ottawa Scale

Study	Selection			Comparability		Outcome			Score
	Case selection	Representativeness of exposed cohort	Representativeness of non-exposed cohort	Comparability of baseline data	Comparability of ER vs SH	Assessment of outcome	Follow-up	Adequacy of follow-up	
Zhou C	1	1	1	1	2	1	1	1	9
Wu JJ	1	1	0	1	2	1	1	0	7
Zhong F	1	1	1	1	2	1	0	1	8
Yang H	1	1	1	1	1	1	0	0	6
Ren A	1	1	0	1	2	1	1	0	7
Ou D	1	1	1	1	2	1	1	1	9
Hsueh KC	1	1	1	0	2	1	0	1	7
Buczkowski AK	1	1	0	1	2	1	0	1	7
